# Influence of Sintering Temperature on the Microstructure and Mechanical Properties of In Situ Reinforced Titanium Composites by Inductive Hot Pressing

**DOI:** 10.3390/ma9110919

**Published:** 2016-11-11

**Authors:** Cristina Arévalo, Isabel Montealegre-Meléndez, Enrique Ariza, Michael Kitzmantel, Cristina Rubio-Escudero, Erich Neubauer

**Affiliations:** 1 Department of Engineering and Materials Science and Transportation, School of Engineering, University of Seville, Camino de los Descubrimientos s/n, Seville 41092, Spain; imontealegre@us.es (I.M.-M.); enrarigal1@hotmail.com (E.A.); 2 RHP-Technology GmbH, Forschungs und Technologiezentrum, Seibersdorf 2444, Austria; michael.kitzmantel@rhp-technology.com (M.K.); erich.neubauer@rhp-technology.com (E.N.); 3 Department of Computer Languages and Systems, University of Seville, Avenida Reina Mercedes s/n, Seville 41012, Spain; crubioescudero@us.es

**Keywords:** titanium matrix composites, boron carbide, Ti_x_Al_y_ intermetallic, inductive hot pressing

## Abstract

This research is focused on the influence of processing temperature on titanium matrix composites reinforced through Ti, Al, and B_4_C reactions. In order to investigate the effect of Ti-Al based intermetallic compounds on the properties of the composites, aluminum powder was incorporated into the starting materials. In this way, in situ Ti_x_Al_y_ were expected to form as well as TiB and TiC. The specimens were fabricated by the powder metallurgy technique known as inductive hot pressing (iHP), using a temperature range between 900 °C and 1400 °C, at 40 MPa for 5 min. Raising the inductive hot pressing temperature may affect the microstructure and properties of the composites. Consequently, the variations of the reinforcing phases were investigated. X-ray diffraction, microstructural analysis, and mechanical properties (Young’s modulus and hardness) of the specimens were carried out to evaluate and determine the significant influence of the processing temperature on the behavior of the composites.

## 1. Introduction

Titanium Matrix Composites (TMCs) are advanced materials made of titanium or titanium alloys mixed with continuous or discontinuous reinforcements. These types of materials exhibit excellent properties such as corrosion and temperature resistance, high specific stiffness, and high Young’s modulus [1,2,3,4].

Among all the proposals for reinforcement categories, the ones that are distributed in a discontinuous way have captured the most attention since they can improve material efficiency at lower cost. However, this involves certain difficulties such as obtaining a clean interface matrix/particle, because in TMCs, numerous reinforcements are unstable and react to form undesired secondary phases. Therefore, it is necessary to understand how the type of reinforcement, the manufacturing method, and certain thermal treatments may affect the physical and mechanical properties of composite materials [5,6].

Concerning the selection of the reinforcing phases, they need to have high hardness, high temperature resistance, high stiffness, thermodynamic stability with titanium and its alloys at sintering temperature, and insolubility in a titanium matrix.

Regarding reinforcement materials, boron carbide (B_4_C) has been presented as a promising ceramic material to provide high hardness, and good wear and corrosion resistance [7]. Recent investigations have been centred on the use of titanium and boron carbide powder for in situ TMC manufacturing, because it is possible to obtain titanium boride (TiB_x_) and titanium carbide reinforcements that have excellent properties, such as high hardness and temperature resistance [8,9,10,11,12,13].

In previous works, the addition of aluminium to form the intermetallic compounds (Ti_x_Al_y_) was investigated [14,15,16]. Developments in the synthesis of Ti_x_Al_y_ by rapid sintering routes studied the formation of various intermediate intermetallic compounds from elemental Ti, Al metals by solid-state reactive diffusion, and involved multiple reaction steps [17]. These intermetallic compounds are particularly attractive for high temperature structural applications, because of improvements in their mechanical properties and high corrosion resistance of the materials. These intermetallic compounds have properties that lie between those of metals and ceramics [18,19].

Powder metallurgy has provided solutions for fabricating TMC materials, since it has greater versatility than other manufacturing methods. The ability to properly choose the constituents of the material is an important and attractive advantage over traditional manufacturing processes such as machining or casting. Moreover, the flexibility in the selection of the fabrication parameters such as processing temperature, consolidation pressure, holding time, and heating rate lead to the modification of the microstructure and the behaviour of the produced specimens, in order to achieve desirable properties [3,6,20,21,22].

The main contributions of this paper are the following: (i) Manufacturing of TMCs by inductive hot pressing at different temperatures, in order to evaluate the effect of this processing parameter on the final material properties; (ii) Studying the influence on the microstructure of in-situ secondary phases from the addition of B_4_C and Al to form Ti-Al as an intermetallic compound; (iii) Relating microstructural changes due to the processing temperature to the hardness and Young´s modulus of the materials have been evaluated.

## 2. Materials and Methods

**Figure 1:**
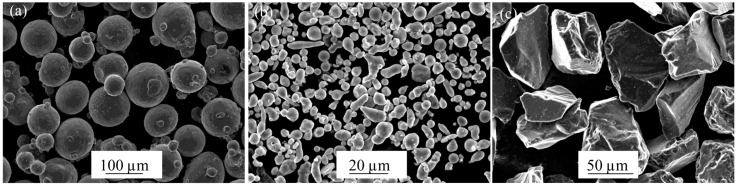
SEM images of the raw powders; (**a**) spherical titanium matrix powder morphology; (**b**) blended powders of fine titanium and aluminium particles; (**c**) irregular and angular B_4_C particles.

The starting powder materials are an important topic of study in the analysis of composite materials. In this work, a spherical titanium matrix powder (grade 1), manufactured by AP&C (Quebec, QC, Canada), was used. To form the reinforcing phases, boron carbide particles were selected (manufactured by ABCR GmbH & Co. KG, Karlsruhe, Germany). Moreover, the presence of in-situ Ti_x_Al_y_ based intermetallic was a main goal of this research. For this reason, elementary aluminium and fine titanium powder were used as raw materials. In this way, this raw materials blend led to an expected Ti-Al intermetallic (manufactured Ti powder by TLS and Al powder by NMD GmbH, Laufen, Germany) [19]. One of the main advantages of using mixed powders instead of prealloyed powders is improved densification. In order to achieve a complete Ti-Al reaction to form intermetallic compounds, Ti fine powder was selected. Hence, the small particle size powder had a high diffusion rate, which was beneficial to the Ti-Al reaction. The different morphologies of the starting powder materials are shown in Figure 1. Characterization of all the raw materials was performed to verify the information supplied by the manufacturer about the size and morphology of the powders (see Appendix A). 

### 2.1. Experimental Procedure

This section describes the different stages of the experimental procedure. Firstly, the powder preparation and consolidation took place. Then, a complete characterization was performed for all the specimens studied. Microstructural analyses were carried out by X-ray diffraction, optical microscopy (OM), scanning electron microscopy (SEM), and energy dispersive spectroscopy (EDS), to examine the microstructural evolution versus variations of the processing temperature. Furthermore, density, Young’s modulus, and hardness of the samples were measured.

#### 2.1.1. Thermodynamic Analysis

Recent research publications ([23,24,25]) have investigated the reactions in the Al-Ti-B_4_C system, the calculation of the reaction directions and the expected stable phases, and their compositions at high temperatures. These studies have been taken into account for understanding the in-situ reaction mechanism that can occur in the Al-Ti-B_4_C system and between the reactants and some intermediate phases.

#### 2.1.2. Powder Preparation

For the starting powder preparation, the Ti-Al intermetallic blending was made with 64% weight percentage of fine titanium powder and 36% weight percentage of aluminium powder. It was developed according to the atomic ratio Ti:Al. This first powder mixing was carried out in a Sintris mixer for 12 h, and using ZrO_2_ balls (Ø 3 mm) in cyclohexane. The weight ratio of ceramic balls to powder was 10:1. After the drying of the Ti-Al mixture in a vacuum oven at 100 °C for 6 h to evaporate the solvent, the powders were blended for 2 h.

Once the Ti-Al powder was prepared, the mixing of the starting materials of the composite was performed. The titanium composites were made of spherical titanium matrix powder (50 vol %), 30 vol % of B_4_C particles, and 20 vol % of the blended Ti-Al powders. All these particulate materials were also blended in a Sintris mixer for 2 h.

#### 2.1.3. Inductive Hot Consolidation Process

The consolidation of the specimens was performed in a self-made hot pressing machine, using inductive Hot Pressing (iHP) equipment from RHP-Technology GmbH & Co. KG, Seibersdorf, Austria. Its main advantage is its high heating rate due to its special inductive heating set up. The die used for all the iHP cycles was produced from graphite (punch Ø 10 mm). For each iHP run, the die was lined with thin paper with a protective coating of boron nitride (BN). The fixed processing parameters were: compaction pressure (40 MPa), holding time (5 min), heating rate (50 °C·min^−1^), and vacuum condition (10^−5^ mbar). Since the effect of the processing temperature was the major topic of this research, the temperatures studied were 900 °C, 950 °C, 1000 °C, 1100 °C, 1200 °C, 1300 °C, and 1400 °C.

#### 2.1.4. Specimen Characterization

When the iHP cycles were finished, the samples were removed from the die and cleaned by a sand blasting machine to remove the graphite paper rests from the surfaces. The specimens produced by iHP offered maximal diameters of 10 mm. 

Microstructural analysis consisted of the study of the specimens’ microstructures by the use of OM (Nikon Model Epiphot 200, Tokyo, Japan) and SEM (JEOL 6460LV equipped with EDS, Tokyo, Japan). Before carrying out the microstructural study of the specimens, they were thoroughly metallographically prepared. Then, X-ray diffraction (XRD) studies were carried out using a Bruker D8 Advance A25 (Billerica, MA, USA), to identify the diverse crystalline phases in the composites.

Then, the measure of the densification of the composites took place. The Archimedes’ method (ASTM C373-14) was used for density determination. The results obtained were compared to the measurements performed with other control techniques such as geometrical density (ASTM B962-13). Moreover, the densification of the specimens was estimated by image analysis using the software Image-Pro Plus 6.2 (Media Cybernetics, Rockville, MD, USA).

A tester model, Struers-Duramin A300 (Ballerup, Germany), was used to ascertain the Vickers hardness (HV10). On the polished cross-section of the specimens, the hardness measurements were carried out. Three indentations were performed on each specimen, avoiding B_4_C particles.

The Young’s Modulus was estimated with the ultrasonic method (Olympus 38 DL, Tokyo, Japan). It was performed with a pulse generator/receiver, recording the transit time (outward/return) through the thickness. This method allowed us to determine both the longitudinal (VL) and transverse (VT) propagation velocities of the acoustic waves. To correctly measure the propagation velocities of waves, the surface of the samples must be properly grinded and polished (samples with smooth and parallel surfaces), and the delay times of transducers must be minimized by following an iterative measurement protocol. The Young’s Modulus was calculated from the density (g/cm^3^), VL, and VT [26].

## 3. Results and Discussion

After characterization of the specimens, the results were evaluated, compared, and discussed while taking into account the effect caused by the increase of the processing temperature.

### 3.1. Microstructural Analysis

A detailed microstructural analysis was conducted to evaluate the influence of the processing temperature on the microstructure of the specimens. Not only was microstructure analysis by optical and electronic microscopy employed, but also X-ray patterns and EDS spectra were used to study the microstructural variations of the specimens.

**Figure 2:**
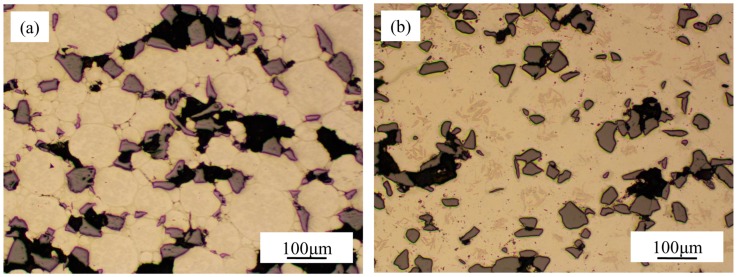
Optical microscopy images of specimens produced at (**a**) 900 °C and (**b**) 1400 °C.

Images obtained under OM of the samples produced at the lowest (900 °C) and the highest (1400 °C) processing temperatures are presented in Figure 2. Even without etching taking place, grain boundaries of titanium matrices were slightly visible and could be identified from 900 to 1100 °C. However, at higher temperature they were not visible, observing dark smaller areas inside the matrix grains.

**Figure 3:**
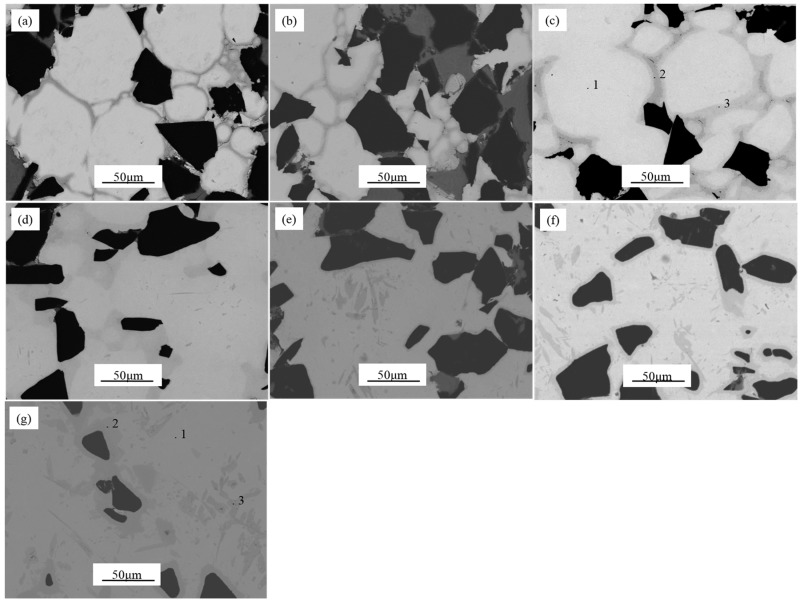
SEM images of the specimens produced at different processing temperatures: (**a**) 900 °C; (**b**) 950 °C; (**c**) 1000 °C; (**d**) 1100 °C; (**e**) 1200 °C; (**f**) 1300 °C and (**g**) 1400 °C.

The higher the temperature was set, the smaller the porosity was detected; it was confirmed by image analysis (see below). This result was expected since the temperature helped the densification and promoted the diffusion phenomenon. With regard to the location of the pores, their presence was observed close to the B_4_C particles. This fact occurred in all the microstructures of the specimens observed. Figure 3 shows SEM images of the specimens produced at different processing temperatures. A clear evolution of the specimens’ microstructures can be appreciated. Amongst the different areas observed, the regions bordering the matrix grains are a feature to highlight. Furthermore, different grey tonalities could be softly spotted. Up to 1000 °C, the matrix grain could be identified due to the grey borders. However, these grey regions became less recognizable increasing the processing temperature, as it has been seen in Figure 3 [27,28].

In the temperature range from 1100 °C to 1300 °C, the grey area observed at the grain boundaries presented less intensity and lightly blurred color. In contrast, there were new dark grey areas located around the B_4_C particles and in the matrix. These areas became more recognizable as temperature increased up to 1400 °C. The reaction between the titanium matrix and the B_4_C particles at that temperature promoted the formation of the reinforcing in situ TiC and TiB based compounds. The well-known morphology of needle shaped TiB and round shaped TiC could be appreciated in Figure 3 for specimens produced at high temperature [22].

In order to determinate the elements in these distinct regions, EDS analyses were carried out in specimens produced at 1000 °C and at 1400 °C. The spots analyzed can be observed in Figure 3c,g. EDS analyses revealed that there was TiB formed around B_4_C particles as well as in the needle form.

**Figure 4:**
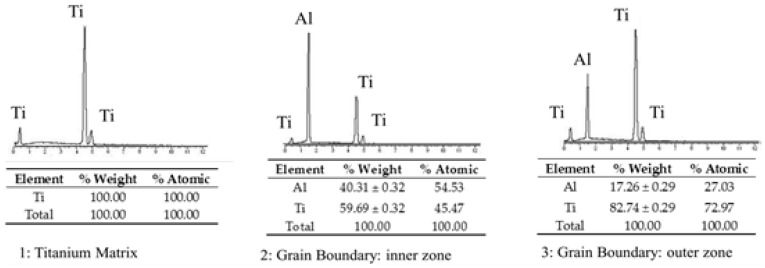
EDS analyses in principal spots at 1000 °C.

The distribution of Al in the matrix can be affected by the increase of the processing temperature. The EDS analyses in specimens produced at 1000 °C revealed Al presence in the grain boundaries observed as grey borders (see in Figure 3c). Predictably, such regions could be the in situ formed Ti-Al intermetallic compounds. These results of the three measured spots have been shown in Figure 4. XRD analysis confirmed this phenomenon. These results are in good agreement with a previous research work in which the synthesis of Ti_x_Al_y_ from elemental Ti-Al powders were obtained via SPS technique at similar temperature [17]. 

**Figure 5:**
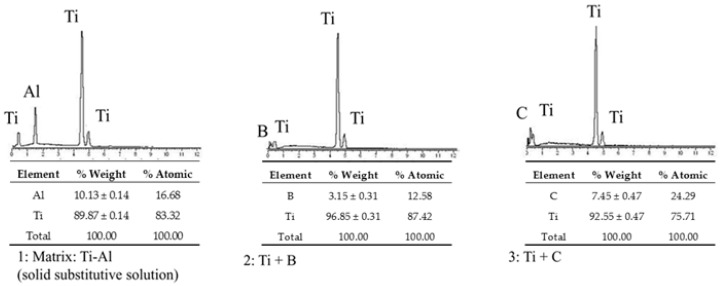
EDS analyses in principal spots at 1400 °C.

Due to the melting point of aluminium being significantly different to titanium and the possible reaction between both elements, we expected a variation of Al located in the microstructure of the specimens produced at higher temperatures. To verify such a phenomenon in specimens fabricated at 1400 °C, three visible regions were also analyzed by EDS. In Figure 3g and Figure 5, it can be seen that at this temperature, Al was detected as a solid substitutive solution with Ti.

With respect to the reaction between the titanium matrix and reinforcement particles (B_4_C), in the specimens produced at temperatures lower than 1000 °C, the presence of TiC and TiB was not detected by EDS analysis (see in Figure 4). However, as it might be expected, samples produced at higher temperatures promoted the formation of TiC and TiB. Contents of B and C were slightly detected in the spot 2 and spot 3 (see Figure 3g and Figure 5). These measurements could give qualitative information about the evolution of the microstructure of the specimens in relation to temperature.

**Figure 6:**
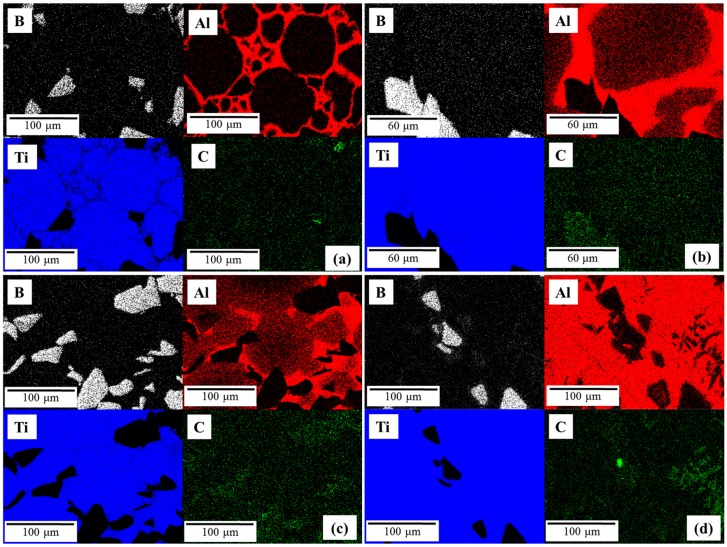
EDS mappings at different manufacturing temperatures: (**a**) 900 °C; (**b**) 1000 °C; (**c**) 1100 °C and (**d**) 1400 °C.

EDS mappings of the specimens produced at 900 °C, 1000 °C, 1100 °C, and 1400 °C were performed qualitatively. They have been reported in Figure 6 in which an overall view of the distribution within the specimens of the described elements (Ti, Al, B, and C) have been shown. Figure 6a–c confirmed that, at lower temperature, B was located exclusively in the B_4_C particles. However, at 1400 °C, B was detected not only in these particles but also as slight variations in the matrix, related to the TiB based precipitate. 

Regarding the presence of Al, the results described previously were verified by this EDS mapping. Al was forming Ti-Al based compounds at 900 °C and 1000 °C, while an increase of the processing temperature led Al to diffuse into the matrix. This phenomenon can be observed in Figure 6c,d. For the specimen produced at 1400 °C, Al was distributed in the matrix, but there was no presence of Al close to the B_4_C particles in some areas of the matrix, due to the precipitation of TiB and TiC (Figure 6d). 

In the same way, the distribution of Ti was affected by the processing temperature. As a matrix element, Ti was easily identified in the matrix grain in Figure 6a. Moreover, the possible reaction with Al to form Ti-Al based intermetallic led to recognition of Ti close to Al, as it can been seen in Figure 6b. At the highest processing temperature, Ti was detected in almost all of the specimens except in the center of B rich areas. Such areas were the B_4_C particles, which did not react completely with the matrix. 

The distribution of C was not useful, since impurities from the metallography preparation were detected. It caused difficulties in identifying the possible TiC compounds. Only in specimens produced at 1400 °C, were there several areas in which the presence of C was remarkably caused by in situ formed TiC.

### 3.2. X-ray Diffraction

**Figure 7:**
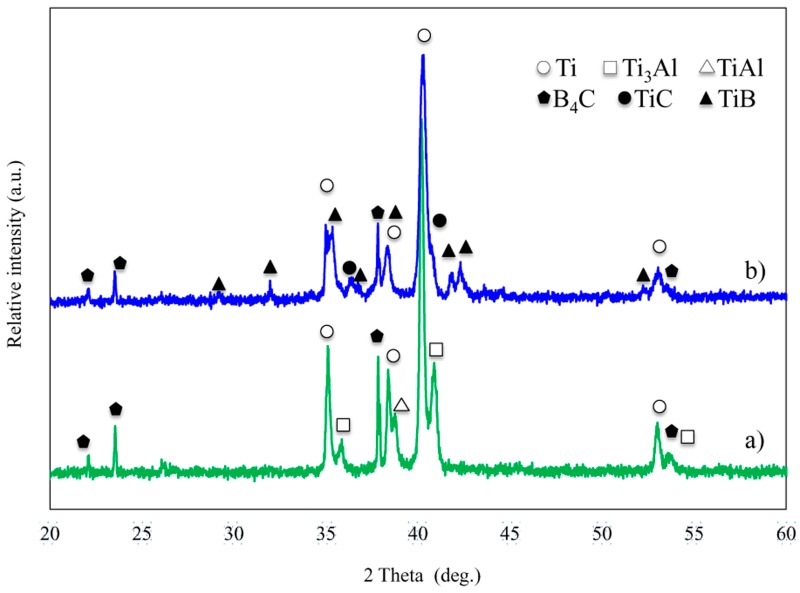
XRD patterns of the Ti matrix with 20 vol % of TiAl and 30 vol % of B_4_C produced during 5 min via inductive hot pressing at different temperatures: (**a**) 1000 °C; and (**b**) 1200 °C.

The evolution of the in situ formed phases with processing temperature in the composites was further studied by XRD. Particular attention was paid to samples produced at 1000 °C and 1200 °C. These temperatures were considered relevant to evaluate the solubility of Al in Ti, in order to study the phase transformation and the solid solubility of this system. The XRD patterns at these two processing temperatures are shown in Figure 7. As observed, the pattern of the specimen produced at 1000 °C displayed sharp Bragg diffraction peaks of Ti and B_4_C with high intensity. However, based on the thermodynamic considerations discussed in previous works [29], the solid-state reaction between Ti and B_4_C to form TiC and TiB based compounds was the most favourable reaction, but was also very slow kinetically. The processing time was 5 min for all cases, and the temperature was the factor responsible for the changes in the microstructure. No formation of TiC and TiB were observed either in XRD or microscopy. In addition to Ti and B_4_C peaks, TiAl and Ti_3_Al peaks were identified [30,31]. Therefore, no reaction between Ti and B_4_C took place at 1000 °C but between Ti and Al to form TiAl and Ti_3_Al. Studying previous works, in which B_4_C-Al systems were researched, B_4_C was moderately absorbed by aluminium; however in the present work, the formation of any Al-B_4_C based compounds was not identified [32,33,34]. 

It is worth noting that, despite 5 min of processing time, the increase of the temperature up to 1200 °C involved a reaction between the reinforcing particles and the matrix. The peaks of the reaction products, TiB and TiC, appeared with low intensity, as shown in Figure 7. Relevant peaks of Ti and B_4_C were also seen. It means that an incomplete reaction between C and Ti, and B and Ti took place. This pattern was in good agreement with the results from the SEM and EDS characterization. According to previous studies [23], TiB_2_ peaks were not detected since this phase transformation requires a very long time. Meanwhile, the peaks of the intermetallic TiAl and Ti_3_Al were not visibly detected. By increasing the temperature, the area under the peaks of Ti changed. A shift to a higher angle in the Ti main peak in the diffraction pattern indicated that Al diffused in the Ti matrix via solid state diffusion. Based on these results, SEM images, and EDS mapping, it could be confirmed that Al was a solid solution in Ti at this temperature.

### 3.3. Densification, Hardness, and Young’s Modulus

**Figure 8:**
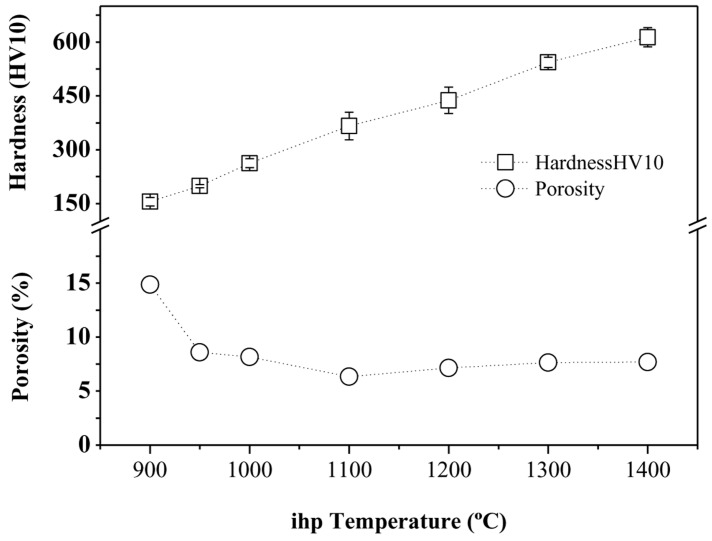
Hardness (HV10) and porosity (%) vs. iHP temperature (°C).

By increasing the temperature, variations of densification and hardness of the specimens were expected (see Figure 8). Due to the short processing time (5 min), any temperature increment significantly enhanced the composites’ densification. In Figure 8, we can observe the trend of hardness (HV10) and porosity (determined by image analysis) of the specimens versus the iHP processing temperature. As is well-known, as the processing temperature increased, better specimen densification was obtained. Hardness behavior was also determined by an increase with processing temperature.

Regarding the specimens’ densification, there was a significant decrease in porosity, by 42.2%, when increasing the iHP temperature from 900 to 950 °C. In such a temperature range, the hardness of the specimens enhanced by up to 28%. An additional 50 °C increase of the processing temperature (from 950 to 1000 °C) contributed to the production of specimens with much better densification properties, reducing the porosity by 8.6% and increasing the hardness by 32.2%. With iHP temperature increments of 100 °C, from 1000 °C, the densification of the specimens increased slightly (see Table 1), while there was a remarkably trend of enhanced hardness. These results can be related to the reactions confirmed between the titanium matrix and the reinforcement materials. The apparitions of new reinforcing phases were promoted by the increase of the processing temperature. These reinforcing phases not only contributed to improve the densification, but also increase the hardness. The maximum densification reached was ~95%. There are several factors justifying these porosity values. Firstly, due to the different diffusion velocities of Ti and Al, poor densification of elemental Ti-Al alloys could be expected. In addition, small cracks are detected at grain boundaries when intermetallic compounds are formed; this phenomenon is observed mostly at low processing temperature values (900–1000 °C). Furthermore, the interaction between the particles of B_4_C (30 vol % content) and the matrix caused a reduction of the densification. The agglomeration of B_4_C particles could have promoted the apparition of porosity between particles. However, an increment in temperature moderates this effect. In order to improve densification results, several holding times, as well as compaction pressure values, should be studied and tested.

Concerning the Young’s modulus calculated, a similar tendency was observed. Specimens manufactured at the highest temperature showed greater Young’s modulus values, and thus also the best specific stiffness. It was a consequence of the promotion of new phases, TiC and TiB formed in the matrix at high temperature. This phenomenon had been verified previously by XRD and microscopy. Thanks to the formation of TiB and TiC as new phases distributed inside the matrix, enhancement of hardness and stiffness in these TMCs took place. This is in agreement with the Orowan strengthening mechanism in metal matrix composites [35]. A higher evolution in properties from the transition temperatures was demonstrated (1200 °C). Young modulus´s values described in the literature are similar or even lower than the ones measured in this work [36,37].

## 4. Conclusions

In this work, the processing temperature was investigated as an influencing factor in the behavior of titanium based composites which were made of 20 vol % of Ti-Al and reinforced with 30 vol % of B_4_C particles. The properties of the specimens were evaluated in terms of microstructure and phase formation in addition to densification, hardness, and Young’s modulus. The following conclusions can be drawn:

- The microstructure of the composites was significantly affected by the processing temperature. In terms of microstructure change, an inflection change was observed between 1000 and 1200 °C. Whilst the matrix grains were visibly defined by the location of TiAl and Ti_3_Al at 900 °C, 950 °C, 1000 °C and less pronounced at 1100 °C, this phenomenon was not demonstrated in composites fabricated at higher temperatures due to the solubility of Al in Ti. Furthermore, the microstructure of the composites essentially evolved from the reaction of Ti and B_4_C.
- Based on the XRD results, it can be suggested that the in situ TiC and TiB compounds were not formed up to 1200 °C, since this reaction was incomplete. Even at 1400 °C, such a reaction was not finished since smaller B_4_C particles were observed in the microstructural analysis.
- By increasing the processing temperature, the hardness of the composites increased (30% for each 100 °C temperature increment), while the density increased slightly. The Young’s Modulus exhibited a similar trend, which increased by 20% at 1000 °C and gradually enhanced by 10% from there on.

## Figures and Tables

**Table 1:** Geometrical and Archimedes’ density, Young’s modulus, and specific stiffness of specimens manufactured at different iHP temperatures (from 900 to 1400 °C).

Manufacturing Temperature (°C)	Geometrical Density, ρ (g/cm^3^)	Archimedes’ Density, ρ (g/cm^3^)	Young’s Modulus, E (GPa)	Specific Stiffness, E/ρ (GPa/g·cm^3^)
900	3.51	3.53	113	37.01
950	3.50	3.62	138	38.12
1000	3.56	3.65	164	44.93
1100	3.53	3.66	167	45.62
1200	3.59	3.71	186	50.13
1300	3.73	3.81	211	55.38
1400	3.80	3.88	237	61.08

## Appendix A

The particle size distributions of the starting powder materials were measured by laser diffraction analysis (Mastersizer 2000). All the scanning electron microscopy (SEM) images of the powders were taken using a JEOL 4640LV. The results of the particle size measurements are listed in Table A1. The d_50_ (median) has been defined as the diameter where half of the population lies below this value; 90 percent of the distribution lies below the d_90_, and 10 percent of the population lies below the d_10_.

**Table A1:** Particle size distributions of the starting powders.

Material	d_10_ (μm)	d_50_ (μm)	d_90_ (μm)
Ti (matrix)	84.70	116.74	162.37
B_4_C	39.95	63.31	98.68
Fine Ti	11.88	28.13	51.42
Al	2.61	6.12	14.87

The supplied information about the starting powders was verified by SEM images, as can be seen in Figure 1. The matrix titanium powder had a spherical morphology confirming the supplier’s information. The highest density is expected to be associated with small spherical particles with hard surface. The SEM image of the blending of fine titanium and aluminium powders reflected the fine particles’ sizes and the round morphologies of both powders. Figure 1c shows the uniform and faceted morphology of B_4_C ceramic particles.
